# Risk factors for nephrolithiasis formation: an umbrella review

**DOI:** 10.1097/JS9.0000000000001719

**Published:** 2024-05-29

**Authors:** Yucheng Ma, Chao Cheng, Zhongyu Jian, Jun Wen, Liyuan Xiang, Hong Li, Kunjie Wang, Xi Jin

**Affiliations:** Department of Urology, Institute of Urology (Laboratory of Reconstructive Urology), West China Hospital, Sichuan University, Chengdu, People’s Republic of China

**Keywords:** kidney stone disease, nephrolithiasis, risk factors, umbrella review, urology

## Abstract

**Objective:**

Nephrolithiasis is prevalent and burdensome worldwide. At present, evidence on the risk factors for nephrolithiasis is unconsolidated and the associations remain uncertain. The authors systematically evaluate the robustness of the meta-analytic evidence and aid more reliable interpretations of the epidemiological relationships.

**Methods:**

The authors conducted a comprehensive review of the meta-analyses, screened the included studies with the aid of the AMSTAR 2 evaluation tool, and then used R (4.1.1) software to perform data analysis to evaluate the association between candidate risk factors and kidney stones, and evaluated the credibility of the evidence of the association between risk factors and kidney stones according to the GRADE classification, and finally obtained the strength and effectiveness of the association.

**Results:**

The authors finally included 17 meta-analyses regarding 46 risk factors, 34 of which (73.9%) showed statistically significant association with nephrolithiasis. Among the significant associations, the authors found that waist circumference, BMI, dietary intake and fructose intake were positively correlated with the occurrence and development of nephrolithiasis. Caffeine, dietary fiber and DASH-diet showed a tendency to reduce kidney stones. Interestingly, calcium supplementation, dietary calcium, and vitamin D, which are widely believed to be responsible for stone formation, made no difference or even reduced the risk of nephrolithiasis.

**Conclusions:**

The authors’ study demonstrates the suggestive causal (central obesity, type 2 diabetes, gout, dietary sodium, fructose intake and higher temperatures) risk factors of nephrolithiasis. The authors also demonstrate the suggestive causal (coffee/alcohol/beer intake, dietary calcium and DASH-diet) protective factors of nephrolithiasis. To provide epidemiological basis for the treatment and prevention of nephrolithiasis.

## Introduction

Highlights

- The first paper presents a more systematic umbrella review of risk factors and prevention factors for kidney stone disease, and a re-review of published meta-analyses results in a more credible conclusion.
- Central obesity, type 2 diabetes, gout, dietary sodium, fructose intake, and high temperature have been shown to be potential risk factors for kidney stones.
- Coffee/alcohol/beer intake, dietary calcium and DASH-diet can be considered protective factors for kidney stone disease.

Nephrolithiasis, as a disease with a high incidence and recurrence rate, is always troubling people all over the world. In the United States, ~10.6% of men and 7.1% of women suffer deeply from nephrolithiasis, which is comparable to diabetes (9.7%)^1–3^. In China, the incidence in men and women is estimated to be 6.5% and 5.1%, respectively^4^, which is also non-negligible. The presence of nephrolithiasis can lead to more than short-term pain and infection. Progressive hypertension^5^, chronic kidney diseases, and even end-stage kidney failure^6,7^ will be the ultimate outcomes of nephrolithiasis without effective intervention and treatment. Unfortunately, despite surgery or medication, recurrence rates of 50% within 5 years and up to 75% within 20 years leave many patients suffering^8^. Meanwhile, the medical expenses incurred by patients due to nephrolithiasis crush their economic lives either. As early as 2005, the annual medical cost of nephrolithiasis in the United States had already exceeded $5 billion^9^. Taking into account the continued growth of people at risk of obesity and diabetes, it is estimated that the cost could increase by $1.24 billion per year until 2030^10,11^.

In the face of such intractable disease, scientists have proposed a series of measures to prevent kidney stones based on epidemiological investigations and statistics, aiming at relevant risk factors, such as increasing fluid intake, supplementing citrate and limiting animal protein intake^12^. Nonetheless, the enhancing incidence of nephrolithiasis can still be observed around the world^13^, which reflects that the summary of the risk factors for the onset of nephrolithiasis is not comprehensive enough and needs to be refined and improved.

Umbrella review, also known as systematic review of systematic reviews, is a research method that systematically evaluates all systematic reviews and meta-analyses on a specific medical research topic to obtain more reliable conclusions^14,15^. Umbrella reviews not only help researchers save time and effort from starting from scratch but also provide a bird’s eye view and recommendations on how certain medical phenomena are associated with exposure to related risk factors^16^.

To date, there have been numerous systematic reviews and meta-analyses on the pathogenesis of nephrolithiasis, but their methodological quality and the quality of the evidence still need to be further validated and evaluated. Therefore, this article aimed to provide an umbrella review of published systematic reviews and meta-analyses to obtain the available evidence on risk factors associated with nephrolithiasis and to provide guidance for the prevention of nephrolithiasis.

## Methods

The umbrella review was registered with PROSPERO. Detailed methods for umbrella review were demonstrated in the Supplementary materials 1, Supplemental Digital Content 1, http://links.lww.com/JS9/C677. Moreover, this research evaluated the effect of kinds of factors on nephrolithiasis formation and followed the Preferred Reporting Items for Systematic Reviews and Meta-analysis (PRISMA) Guideline^17^, see in Supplementary Fig 1, Supplemental Digital Content 2, http://links.lww.com/JS9/C678. Besides, according to the World Medical Association’s Declaration of Helsinki in 2013, this study has taken through the registration process at Research Registry (http://www.researchregistry.com).

### Literature search strategy, inclusion and exclusion criteria, and data extraction

The PubMed, Embase and the Cochrane library databases were searched by two independent researchers from inception to 1 November 2021, with English language restrictions, to identify studies. The detailed search terms are provided as follows. We included meta-analyses that explored the association between any risk factor and nephrolithiasis in the observational study. We excluded systematic reviews without meta-analysis. The first author, publication year, number of cases and participants, risk factor of interest, estimate with its 95% CI from the largest primary study, metrics used for pooling analyses, and study design were the main information that was extracted. Other information based on the random-effects model, including I^2^, tau^2^, Z value, and the *P* value of Egger’s test, was also collected to verify the subsequent re-analyzed meta-analysis results.

### Search strategy for umbrella review

#### PubMed

The specific search methods used to search the included literature can be found in the Supplementary Materials, Supplemental Digital Content 1, http://links.lww.com/JS9/C677 of this paper. The search strategies used for the other databases were almost identical or slightly modified depending on the circumstances of each database.

#### AMSTAR 2 for the umbrella review

AMSTAR 2 is a critical appraisal tool for systematic reviews that include randomized or non-randomized studies of healthcare interventions. When more than one meta-analysis was included, we extracted information from the meta-analysis with the highest AMSTAR 2 level^18^, the detail checklist could be saw in the Supplementary Fig 2, Supplemental Digital Content 3, http://links.lww.com/JS9/C679.

#### Statistical analysis for the umbrella review

We re-analyzed each eligible meta-analysis using a random-effects model. The following indexes were used to evaluate bias: a small study effect could be recognized if the *P* value of Egger’s test is small of 0.05 or *P* less than 0.1 with the pooling estimate larger than the estimate of its largest component study^19^. The I² statistic was calculated, and high heterogeneity was defined as I² greater than 50%^20^. The 95% prediction interval could predict that the probability of the effect size of each future individual study falling within the prediction interval is 95%^21^. The χ^2^ test was used to detect excess significance bias^22^.

We used a Bonferroni-corrected *P* value to account for multiple testing: *P* less than 0.0016 (0.05 divided by 32) was the significance level, and a *P* value between 0.0016 and 0.05 was considered to be a suggestive association. Statistical analyses and data visualization were achieved by the “meta” packages in R (4.1.1).

#### Credibility assessment and certainty of evidence evaluation

We divided the level of evidence with nominal statistical significance (*P* < 0.05) into four categories that mainly referred to a study that previously proposed a credibility assessment^23^: convincing (class I), highly suggestive (class II), suggestive (class III), and weak (class IV). The evaluation criteria can be found as follows. We assessed the certainty of evidence for associations in the umbrella review under the guidance of the Grading of Recommendations Assessment, Development, and Evaluation (GRADE) approach^24^.

#### Credibility assessment for meta-analysis in umbrella review

We mainly referred to the previously proposed study for credibility assessment^23^. Specific evaluation criteria can be found in the Supplementary Materials 2, Supplemental Digital Content 1, http://links.lww.com/JS9/C677.

## Results

**Figure 1:**
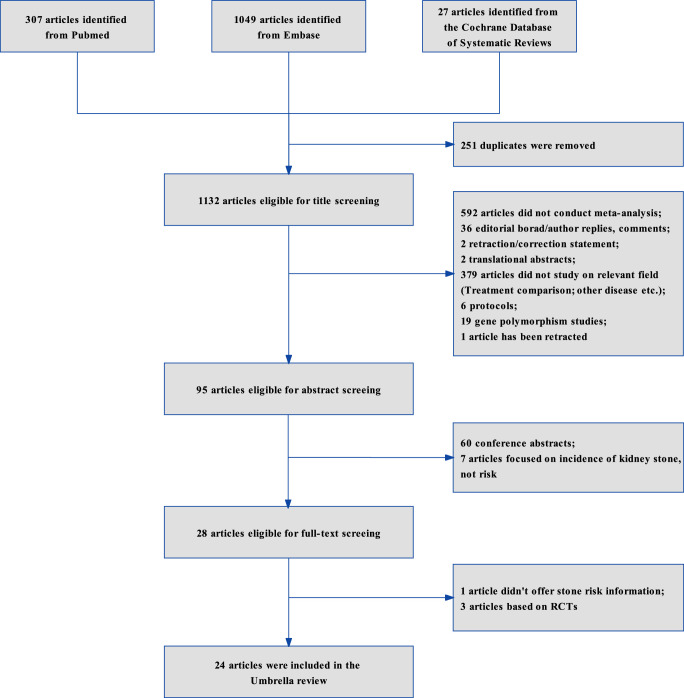
Flow diagram to demonstrate the search and selection process in umbrella review.

Twenty-four articles were included to further assessment after applying the inclusion or exclusion criteria (Fig. 1). Finally, 17 studies on 46 associations were further analyzed by using the AMSTAR 2.0 tool to evaluate all meta-analyses (Supplementary Table S1 and S2, Supplemental Digital Content 1, http://links.lww.com/JS9/C677). All metrics calculated in this umbrella review were the same as those that extracted form original study. In total, 73.9% (34/46) of the associations were statistically significant according to the random-effects model results, of which 10 achieved the *P* less than 1E-06 level. 22 (47.8%) associations had significant heterogeneity (I^2^ > 50%). Obvious flaws (heterogeneity, small study effect, excess significance bias, *P* less than 0.05 of the largest study in meta-analysis) were not detected for 18 (39.1%) associations.

**Figure 2:**
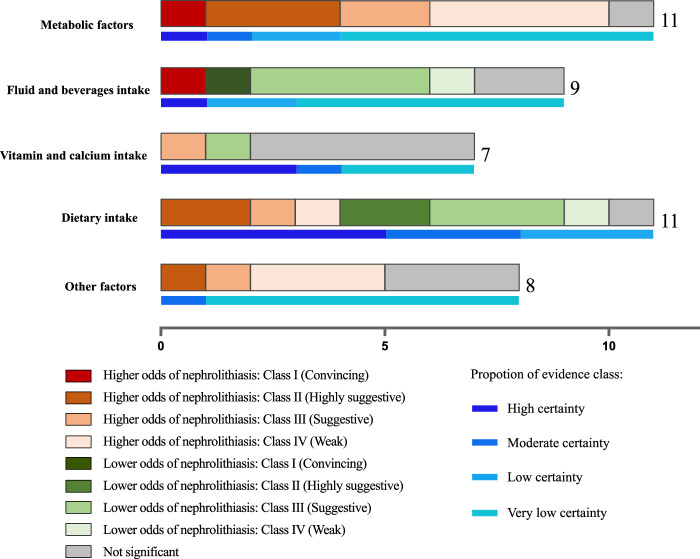
Histogram demonstrating the summary results for number of meta-analyses included and its corresponding class of umbrella review and certain of evidence.

The summary results for number of meta-analyses included and its corresponding class of umbrella review and certain of evidence were demonstrated in Fig. 2. The details of associations between exposures and nephrolithiasis, and evidence class reported in meta-analyses were collected in Table 1.

**Table 1:** Associations between exposures and nephrolithiasis, and evidence class reported in meta-analyses.

Exposures	No. cases/total population	No. of study estimates	Study design	Effect metrics	Effect of largest study in meta-analysis	Random effect summary estimate (95% CI)	Random-effects *P* value	I^2^ (%)	95% prediction interval	*P* value for Egger test	Excess significance bias *P* value	Large heterogeneity, small study effect, excess significance bias, *P*>0·05 of the largest study in meta-analysis	Evidence level
Metabolic factors													
Waist circumference	4282/256 502	3 studies, 5 cohort	cohort	RR	1.18 (1.12, 1.25)	1.16 (1.12, 1.19)	1.63E-19	0	(1.10, 1.20)	0.136	0.731	None	I
BMI	10 368/459 860	5 studies, 8 cohorts	Cohorts/case-control	RR	1.39 (1.26, 1.53)	1.21 (1.12, 1.30)	1.06E-06	77.3	(1.02, 1.90)	0.240	0.141	Large heterogeneity	II
T2D	21 676/738 222	12 studies, 12 cohorts	Cohort/case-control	RR	1.11 (0.91, 1.34)	1.18 (1.07, 1.29)	1.84E-13	60.8	(0.89, 1.57)	0.705	0.401	Large heterogeneity	II
Gout	At least 12 110 cases/NR	3 studies, 3 cohorts	Cross-sectional	OR	1.49 (1.04, 2.14)	1.77 (1.43, 2.19)	1.52E-07	0	(0.44, 7.05)	0.97	0.156	None	II
NAFLD	NR/238 400	8 studies, 8 cohorts	Cohort/Cross-section	OR	1.08 (0.90, 1.30)	1.81 (1.29, 2.56)	6.99E-04	92.1	(0.58, 5.61)	0.737	0.256	Large heterogeneity, *P*>0·05 of the largest study in meta-analysis	III
Metabolic syndrome	At least 1396/64 867^a^	6 studies, 6 cohorts	Cross-sectional	OR	1.11 (1.03, 1.19)	1.35 (1.16, 1.55)	1.77E-04	81.9	(0.80, 2.28)	0.147	0.122	Large heterogeneity	III
HDL-C (decreased)	10 974/257 413	9 studies, 9cohorts	Cohort/Cross-section	OR	0.97 (0.89, 1.06)	1.17 (1.01, 1.36)	0.031	81.2	(0.74, 1.87)	0.319	0.709	Large heterogeneity, *P*>0·05 of the largest study in meta-analysis	IV
Triglycerides	10 272/205 229	8 studies, 8 cohorts	Cohort/Cross-section	OR	1.03 (0.97, 1.10)	1.29 (1.07, 1.54)	0.007	85.9	(0.71, 2.33)	0.098	0.949	Large heterogeneity, small study effect.	IV
Hypertension	At least 18 023/544 713^a^	11 studies, 13 cohorts	Cohort	RR	0.95 (0.91, 1.01)	1.30 (1.11, 1.52)	0.002	92.3	(0.72, 2.36)	0.035	0.435	Large heterogeneity, small study effect, *P*>0·05 of the largest study in meta-analysis	IV
Gallstones	42 084/363 765	2 studies, 4 cohorts	Cohort	RR	1.97 (1.81, 2.15)	1.46 (1.15, 1.85)	0.002	95.7	(0.47, 4.57)	0.424	0.255	Large heterogeneity	IV
Impaired glucose tolerance	At least 1091/62041^a^	4 studies, 4 cohorts	Cross-sectional	OR	1.03 (0.96, 1.10)	1.26 (0.94, 1.58)	0.196	96.1	(0.28, 5.41)	0.971	0.919	Large heterogeneity, *P*>0·05 of the largest study in meta-analysis	NS
Fluid and beverages intake													
Soda	5031/221 728	3 studies, 5 cohorts	Cohorts/case-control	RR	1.51 (1.37, 1.66)	1.38 (1.26, 1.51)	8.49E-12	35.8	(1.08, 1.76)	0.275	0.811	None	I
Caffeine	4982/217 883	1 study, 3 cohorts	Cohorts	HR	0.69 (0.58, 0.82)	0.71 (0.64, 0.79)	8.03E-11	0	(0.36, 1.39)	0.874	0.362	None	II
Fluid	4765/273 954	9 studies, 9 cohorts	RCTs/Cohort/case-control	RR	0.68 (0.56, 0.83)	0.47 (0.33, 0.67)	2.79E-05	90.6	(0.14, 1.58)	0.172	0.37	Large heterogeneity	III
Alcohol	4701/539 583	6 studies, 7cohorts	Cohort/Case-control	RR	0.54 (0.47, 0.62)	0.69 (0.56, 0.85)	2.16E-04	71.9	(0.36, 1.32)	0.296	0.785	Large heterogeneity	III
Beer	6050/223 734	4 studies, 5 cohorts	Cohort/case-control	RR	0.59 (0.46, 0.76)	0.60 (0.49, 0.74)	1.67E-06	35.7	(0.35, 1.04)	0.766	0.547	None	III
Coffee	NR/170 544^a^	6 studies, 10cohorts	Cohort/case-control	OR	0.51 (0.36, 0.75)	0.70 (0.60, 0.82)	1.63E-05	42.7	(0.46, 1.07)	0.439	0.648	None	III
Tea	10 760/790 026	6 studies, 8 cohorts	Cohort/case-control	RR	0.85 (0.78, 0.92)	0.88 (0.79, 0.97)	0.013	63.1	(0.65, 1.19)	0.722	0.697	Large heterogeneity	IV
Milk	At least 6535/224 380^a^	5 studies, 5 cohorts	Cohort/Case-control	RR	1.00 (0.94, 1.07)	0.95 (0.86, 1.05)	0.325	52.9	(0.71, 1.27)	0.652	0.579	Large heterogeneity, *P*>0·05 of the largest study in meta-analysis	NS
Juice	5810/223 102	3 studies	Cohort/Case-control	RR	0.95 (0.88, 1.03)	1.00 (0.92, 1.09)	0.951	33.5	(0.46, 2.17)	0.369	0.748	*P*>0·05 of the largest study in meta-analysis	NS
Vitamin and calcium intake													
Calcium supplement	2087/187 976	2 studies, 2 cohorts	Cohorts	RR	1.13 (0.92, 1.36)	1.16 (1.00, 1.35)	0.047	0	/ (study number not enough)	/ (study number not enough)	0.811	*P*>0·05 of the largest study in meta-analysis	III
Dietary calcium	9695/41 4911	4 studies, 6 cohorts	Cohort	RR	0.79 (0.69, 0.89)	0.83 (0.76, 0.90)	2.43E-05	20.4	(0.69, 0.99)	0.206	0.1425	None	III
Total vitamin D	6905/220 552	2 studies, 4 cohorts	Cohorts	RR	1.18 (0.94, 1.48)	1.07 (0.93, 1.23)	0.358	0	(0.79, 1.46)	0.101	0.715	*P*>0·05 of the largest study in meta-analysis	NS
Vitamin D supplement	7815/210 199	10 studies, 12 cohorts	RCTs/Cohorts/case-control	RR	1.38 (1.03, 1.85)	1.07 (0.92, 1.26)	0.381	0	(0.90, 1.28)	0.359	0.348	None	NS
Total vitamin C	6574/224 272	2 studies, 3 cohorts	Cohorts	RR	0.99 (0.90, 1.09)	1.15 (0.95, 1.26)	0.268	78.0	(0.06, 21.46)	0.491	0.678	Large heterogeneity, *P*>0·05 of the largest study in meta-analysis	NS
Vitamin C supplement	11 917/385 747	5 studies, 8 cohorts	Cohorts/case-control	RR	0.90 (0.79, 1.04)	1.10 (0.95, 1.26)	0.204	64.8	(0.73, 1.66)	0.208	0.583	Large heterogeneity, *P*>0·05 of the largest study in meta-analysis	NS
Total vitamin B6	6905/220 677	2 studies, 4 cohorts	Cohorts	RR	1.06 (0.91, 1.24)	1.01 (0.91, 1.12)	0.881	0	(0.83, 1.27)	0.092	0.681	None	NS
Dietary intake													
Dietary sodium	5172/660 412	4 studies, 4 cohorts	cohort	RR	1.33 (1.12, 1.58)	1.38 (1.21, 1.56)	7.42E-07	23.2	(0.93, 2.05)	0.513	0.449	None	II
Fructose intake	4902/241 538	1 study, 3 cohorts	Cohort	RR	1.27 (1.04, 1.54)	1.33 (1.19, 1.49)	8.65E-07	0	(0.65, 2.74)	0.795	0.194	None	II
Meat	3820/507 780	4 studies, 5 cohorts	Cohorts/case-control	RR	1.21 (1.05, 1.39)	1.24 (1.12, 1.39)	8.49E-05	0	(1.05, 1.46)	0.256	0.131	None	III
Spinach	1473/45 619	1 study, 3 cohorts	Cohort	RR	1.34 (1.10, 1.64)	1.21 (1.01, 1.44)	0.035	56.7	(0.18, 8.07)	0.390	0.266	Large heterogeneity	IV
Fiber	4750/570 859	3 studies, 3 cohorts	Cohort	RR	0.67 (0.60, 0.75)	0.71 (0.64, 0.79)	4.11E-11	15.9	(0.31, 1.63)	0.727	0.223	None	II
DASH-diet	6449/192 126	1 study, 3 cohort	Cohort	RR	0.65 (0.56, 0.76)	0.69 (0.64, 0.75)	6.83E-17	0	(0.42, 1.12)	0.403	0.367	None	II
Dietary potassium	6637/220 677	2 studies, 4 cohorts	Cohort	RR	0.67 (0.57, 0.78)	0.59 (0.46, 0.75)	8.59E-06	75.4	(0.21, 1.65)	0.990	0.725	Large heterogeneity	III
Dietary magnesium	2105/123 956	3 cohorts	Cohort	RR	0.62 (0.43, 0.89)	0.56 (0.46, 0.75)	5.90E-06	0	(0.20, 2.15)	0.06	0.102	None	III
Fruit	6504/588 959	5 studies, 6 cohorts	Cohorts/case-control	RR	0.79 (0.71, 0.89)	0.79 (0.71, 0.89)	1.09E-04	30.1	(0.61, 1.03)	0.527	0.135	None	III
Vegetable	5485/586 953	4 studies, 4 cohorts	Cohorts	RR	0.90 (0.81, 1.00)	0.84 (0.75, 0.94)	0.002	42.7	(0.55, 1.26)	0.695	0.164	None	IV
Energy	8050/350 694	3 studies, 5 cohorts	Cohort	RR	1.01 (0.85, 1.18)	1.12 (0.99, 1.27)	0.075	42.0	(0.26, 4.65)	0.022	0.234	Large heterogeneity, *P*>0·05 of the largest study in meta-analysis, small study effect.	NS
Other factors													
Higher temperatures	At least 490 057 cases/NR^a^	4 studies, 8 cohorts	Cohort/case-control	OR	1.30 (1.20, 1.41)	1.32 (1.24, 1.39)	2.54E-21	0	(1.22, 1.43)	0.175	0.044	Excess significance bias	II
Polycystic kidney disease	128/1368	6 studies, 6 cohorts	Case-controls	RR	1.75 (0.98, 2.64)	1.85 (1.29, 2.64)	6.99E-04	0	(1.11, 3.08)	0.177	0.724	*P*>0·05 of the largest study in meta-analysis	III
Inflammatory bowel disease	222/1624	5 studies, 5 cohorts	Cohort/case-control	RR	5.62 (4.37, 7.24)	3.86 (1.14, 13.03)	0.030	88.4	(0.08, 197.67)	0.835	0.629	Large heterogeneity	IV
Cadmium exposure	NR/88 045^a^	6 studies, 6 cohorts	Cohort/ Cross-sectional study	OR	1.29 (1.01, 1.61)	1.32 (1.08, 1.62)	0.007	58.1	(0.74, 2.35)	0.001	0.114	Large heterogeneity, small study effect.	IV
Pulp Stones	147/284	2 studies, 2 cohorts	Case-control	OR	1.71 (0.90, 3.5)	1.97 (1.22, 3.18)	0.006	0	/ (study number not enough)	/ (study number not enough)	0.495	None	IV
Physical activity	7747/299 358	2 studies, 4 cohorts	Cohort	RR	1.02 (0.85, 1.21)	0.91 (0.73, 1.12)	0.379	89.4	(0.33, 2.54)	0.204	0.884	Large heterogeneity, *P*>0·05 of the largest study in meta-analysis	NS
Bariatric surgery	NR/11 348^a^	4 studies, 4 cohorts.	RCTs/cohorts	RR	1.71 (1.44, 2.04)	1.22 (0.63, 2.35)	0.551	82.8	(0.07, 20.20)	0.443	0.077	Large heterogeneity	NS
Postmenopausal hormone	NR/71101^a^	3 studies, 7 cohorts	RCTs/cohorts/case-control	RR	1.08 (0.87, 1.34)	0.91 (0.72, 1.14)	0.397	74.1	(0.46, 1.81)	0.007	0.274	Large heterogeneity, *P*>0·05 of the largest study in meta-analysis, small study effect	NS

DASH-diet, dietary approaches to stop hypertension-diet; HDL-C, high-density lipoprotein cholesterol; NAFLD, non-alcoholic fatty liver disease; OR, odds ratio; RR, risk ratio; T2D, type 2 diabetes.

a Some original studies did not offer detailed number of participants.

### Metabolic factors

Of the 10 significant associations, 4 (40%) associations achieved highly suggestive or convincing evidence including waist circumference/BMI, type 2 diabetes (T2D) and gout. The same proportion occurred in associations with weak suggestive evidence, which contained high-density lipoprotein cholesterol (HDL-C) on decreased, triglycerides, hypertension and gallstones. The nephrolithiasis associations with non-alcoholic fatty liver disease (NAFLD) and metabolic syndrome were identified as suggestive evidence. No significant evidence was found for the association, impaired glucose tolerance, by evaluation.

### Fluid and beverages intake

Of the 7 significant associations, highly suggestive, convincing, weak evidences were achieved by only 1 (14.3%) association each, which were soda, caffeine and tea, respectively. 4 (57.1%) associations, covering fluid, alcohol, beer and coffee, got suggestive evidence. Milk and juice were recorded as associations covered no significant evidence.

### Vitamin and calcium intake

Only two associations among all associations of vitamin and calcium intake, calcium supplement and dietary calcium, were significant and achieved suggestive evidence. Other five associations like total vitamin D, vitamin D supplement, total vitamin C, vitamin C supplement and total vitamin B6 were deemed to have no significant evidence.

### Dietary intake

Of the 10 significant associations, 4 (40%) associations achieved Class II credibility level including dietary sodium, fructose intake, fiber and dietary approaches to stop hypertension-diet (DASH-diet). Meat, dietary potassium, dietary magnesium and fruit, these 4 (40%) significant associations got Class III credibility level. For significant associations with weak evidence, spinach and vegetables were classified as them. Of all over associations, only one association without significance and no convincing one were observed.

### Other factors

Two associations, higher temperatures and polycystic kidney disease, owned highly suggestive or suggestive evidence among the five significant associations. Other three significant associations embodying inflammatory bowel disease, cadmium exposure and pulp Stones only achieved Class IV credibility level. By analyzing and evaluating data, physical activity, bariatric surgery and postmenopausal hormone were recorded as associations covered no significance.

### Evaluation of associations by GRADE approach

**Figure 3:**
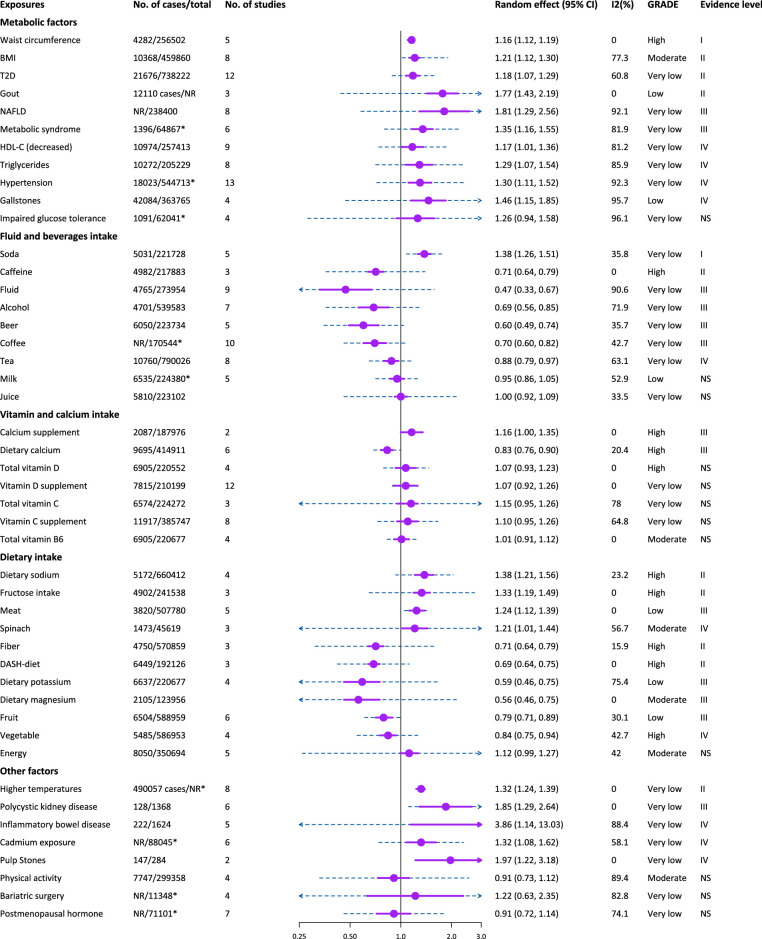
Forest plot to demonstrate the main results of umbrella review. Purple box and solid line are the effect size with its 95% CIs of meta using random-effect model, and blue dotted line represent 95% prediction intervals. 2hGlu, 2-h glucose after an oral glucose challenge; 25(OH)D, 25-Hydroxyvitamin D; HDL-C, high-density lipoprotein cholesterol; NAFLD, non-alcoholic fatty liver disease; T2D, type 2 diabetes.

Of the 46 associations in the umbrella review, we found that only 9 (19.6%) and 7 (15.2%) were supported by high and moderate evidence certainty based on the GRADE approach, respectively (Fig. 3 and Supplementary Table S3, Supplemental Digital Content 1, http://links.lww.com/JS9/C677). This indicated that meta-analyses based on extensive prospective cohort studies for most risk factors are urgently needed in the future.

## Discussion

Our study provided a panoramic display of 46 reported associations from meta-analyses of observational studies between exposures and nephrolithiasis. Of the 34 significant associations, only 11 (32.4%) associations achieved highly suggestive or convincing evidence. Besides, Among the 46 associations, we found that only nine (19.6%) and seven (15.2%) were supported by high and moderate evidence certainty, respectively. Integrating the evidence from the observational studies enables a more reliable interpretation of epidemiological relationships. We found that increased BMI, T2D, higher levels of circulating calcium, urinary calcium, circulating 25(OH)D, and urinary sodium causally increased the risk of nephrolithiasis. Increased waist circumference and waist-hip ratio were suggestively associated with a higher nephrolithiasis risk. In addition, we demonstrated that caffeine and tea intake were closely associated with a lower nephrolithiasis risk. Higher coffee, alcohol, and beer intake, and higher levels of urinary magnesium were suggestively associated with a decreased nephrolithiasis risk.

### Metabolic factors

Metabolic syndrome and its components have been established to be associated with a higher nephrolithiasis risk in observational studies. Besides, obesity/central obesity was supported by high and moderate evidence certainty. Therefore, it would be advisable to conclude an influential relationship between obesity and nephrolithiasis risk. And future similar research is unlikely to change this evidence. In addition, T2D is likely a causal risk factor for the development of nephrolithiasis. A meta-analysis that simultaneously enrolled prospective and retrospective studies simultaneously with high heterogeneity decreased the evidence level (very low certainty). Thus, a prospective updated meta-analysis incorporating a larger number of prospective cohort studies is warranted to increase the level of evidence. The main underlying mechanisms by which obesity and T2D affect kidney stone formation involve urinary derangements, especially a lower urinary pH^25^ and inflammation^26^.

For HDL-C and NAFLD, there is weak evidence that a lower HDL-C level and NAFLD increases the risk of nephrolithiasis. However, neither the primary study with the largest sample nor the only prospective cohort study was not significant. For triglycerides, the results of the largest sample primary study and the only prospective cohort study were inconsistent. Therefore, a meta-analysis based on extensive prospective cohort studies may be required to resolve the disparity between the results of observational studies. Similar to lipid traits, there is weak evidence that hypertension increases the risk of nephrolithiasis, which indirectly suggests the close relationship between abnormal blood lipids and hypertension^27^. On the other hand, gallstone, one of the outcomes of abnormal lipid metabolism^28^, and nephrolithiasis belong to pathological component deposition in the body, but the evidence level for the relevance between the two is very limited. This discrepancy may be partially attributable to the high heterogeneity and small study effect in the meta-analysis of observational studies. Thus, an updated meta-analysis is necessary.

Although gout was found to be associated with nephrolithiasis in observational studies (highly suggestive), we found no formal meta-analysis on serum urate levels and urolithiasis. A cohort study of 239 331 Korean adults reported that increased serum urate levels increased the risk of nephrolithiasis in a dose-dependent manner^29^. In contrast, a recent observational study of the UK Biobank data demonstrated none causal effect of serum urate levels on nephrolithiasis^30^. Therefore, it would be inadvisable to conclude a causal relationship based on the present evidence. Given that nephrolithiasis is a heterogeneous disease that includes, but is not limited to uric acid calculus, the causal effect of serum urate levels could be diluted by other types of urolithiasis, such as calcium oxalate stones. Associations between serum urate levels and gout with urolithiasis should consider the components of stone.

### Fluid and beverages intake

Results of umbrella review demonstrated that caffeine, coffee and tea intake were associated with a lower risk of nephrolithiasis. However, only caffeine reached the highly suggestive class with a high level of evidence certainty. Regarding alcohol and beer intake, the observational studies indicate a suggestive association between them and kidney stone development. However, this association should be treated with caution because the results of partial sensitivity analyses were not significant. The diuretic effects of alcohol, tea, and coffee might be the common mechanism that lowers the risk of nephrolithiasis^31,32^. In addition, tea and coffee might exert many other protective effects to against stone formation, such as caffeine intake, additional fluid volume intake and the effects of antioxidant components^32^. Although taking more coffee, tea, alcohol and beer may help prevent nephrolithiasis, people should balance their potential harms on other organs, especially for alcohol consumption. The long-term impact of over intaking of alcohol on the liver may ultimately involve the formation of nephrolithiasis. Interestingly, the result of Soda in the umbrella review seems to be ambivalent. The attitude that Soda could be the risk factor for nephrolithiasis became whirling due to convincing evidence but poor GRADE-score, which may hint that the key for preventing nephrolithiasis can be caught by clarifying the boundary range of soda intake rather than focusing on the identity definition of Soda. Over the range, soda intake promotes the excretion of calcium, uric acid, and oxalic in the urine^33,34^.

In our review, the effect of juice on the formation of nephrolithiasis was not significant, which may be related to the composition and its influence. In addition to the additional liquid replenishment of juice, orange, lemon, and grapefruit juices are rich in citrate, which plays a role in alkalizing urine in the body^35^, at the same time, the citrate in urine also plays a preventive role in preventing stones^36,37^. On the other hand, the role of orange juice and grapefruit juice in enhancing urine oxalate cannot be ignored^38,39^, and the high carbohydrates and sugars in juice are also risk factors for the formation of kidney stones^40,41^. The role of juice needs to consider not only the influence of multiple components but also the variety of juice, which reflects the need for more specific research. For milk, it is important to note that, contrary to popular beliefs about the relationship between milk and nephrolithiasis, milk does not promote the formation of nephrolithiasis according to our result. Milk, which contains whey protein and albumin, did not affect the average urinary calcium, uric acid, citrate, oxalate, pH, and urinary saturation index in urine^42^.

### Vitamin and calcium intake

Although once reviews demonstrated that higher levels of circulating calcium and urinary calcium were causally associated with kidney stone formation^43,44^, it is crucial to separate genetically predicted higher serum calcium levels and external calcium intake because calcium in the intestine acts as a chelator for oxalate. A low-calcium diet will increase oxalate absorption in the intestine, thereby leading to oxaluria and increasing the risk of calcium oxalate crystal formation. This was supported in the umbrella review that calcium supplement increases, but dietary calcium intake decreases the risk of nephrolithiasis. Neither total vitamin D intake nor vitamin D supplementation was associated nephrolithiasis in the meta-analysis of observational studies. However, our previously published mendelian randomization study^45^ suggested that higher 25(OH)D levels are causally associated with kidney stones. But the effect in the real world depends on the cumulative exposure to vitamin D intervention over time. In theory, long-term extensive supplementation with exogenous vitamin D alone, calcium alone, or a combination of the two to elevate circulating 25(OH)D and calcium levels may increase the risk of nephrolithiasis due to the cumulative effects over time.

In addition to vitamin D, the results of vitamin C and vitamin B6 in our article suggested that there was no significant association with the formation of nephrolithiasis. However, total vitamin C and vitamin C supplementation were observed to be risk factors for kidney stones in men but not in women^46^. This difference may involve hormonal differences between men and women, but the exact mechanism is still not well understood. In the case of vitamin B6, as a cofactor of alanine-glyoxylate aminotransferase, deficiency of vitamin B6 may eventually lead to increased oxalate production and excretion via raising the amount of glyoxylate converted to oxalate through lactate dehydrogenase. Some studies have shown that vitamin B6 intake can reduce oxalate excretion in the urine^47–49^, but other studies have reached contradictory conclusions^50,51^. Reviewing the results we obtained, the impact of vitamin B6 on kidney stones may depend on the degree of intake, high intake may achieve the purpose of stone suppression, but the daily intake of vitamin B6 usually was not paid attention by residents, at the same time, the efficacy of vitamin B6 may also be related to its metabolism in the body.

### Dietary intake

Evidence from epidemiological studies and randomized controlled trials (RCTs) has shown a significant association of sodium intake and urinary sodium levels with urinary calcium excretion^52^. Our study supported a link between urinary sodium levels and nephrolithiasis. The level of urinary sodium reflects the complex interplay between dietary sodium intake and homeostatic mechanisms. Kidney function, potential genetic influence, and other pathways might all contribute to the control of sodium excretion^53^. For patients with high sodium levels in spot urine samples, decreasing dietary salt intake to lower urinary sodium levels would be an effective intervention to reduce nephrolithiasis risk. This view corresponds to DASH-diet in our study, which advocated low in salt, fat and sugar.

Rich in non-dairy animal protein, meat consumption was considered a risk factor for kidney stones because the renal acid load in this diet tends to be inversely proportional to the excretion of citrate in the urine^54,55^, resulting in a negative calcium balance, low citrate, low potassium and low magnesium in the urine, which reduces greatly the ability to inhibit the crystallization of oxalates in the urine^56^. As shown in our article, meat promotes the formation of nephrolithiasis, while dietary potassium and dietary magnesium act as inhibitors. In addition, through animal experiments, meat intake has been verified to affect the changes of gut microbiota. Several studies have found that high-protein diets led to pro-inflammatory changes in the intestines of mice, an increase in disease-causing microorganisms, and a decrease in oxalate-degrading bacteria^57,58^. Other studies have manifested that protein intake, particularly chicken-derived protein, may be associated with positive changes in the gut microbiota of mice, and a significant increase in oxalic-degrading lactobacillus was observed in the gut of mice^59,60^. Differences in these effects may depend on the absolute amount of protein intake, with beneficial flora at an advantage when intake was moderate; When intake was at both extremes, the representation of beneficial bacteria tended to be lowest^61^.

According to our results, dietary fiber can be regarded as an inhibitor of kidney stones, and not surprisingly, fruits and vegetables rich in dietary fiber also exert the same effect. Notably, the effect of fructose intake and spinach contradict the above conclusion. Li and colleagues reported that negatively charged protein biomolecules in spinach leaching solution and calcium ions could biosynthesize calcium oxalate crystals by mutual reaction, which may pave the way for the explanation of the special outcome of spinach.

### Other factors

The increased risk of kidney stones after bariatric surgery has been confirmed by several studies^62–64^, and although this contradicted the results obtained in our study, it also suggests that the results should be understood from multiple perspectives and multi-factorial directions. There are various types of bariatric surgery, including sleeve gastrectomy (SG), biliopancreatic diversion with duodenal switch (BPD-DS) and Roux-en-Y gastric bypass (RYGB); however, only the postoperative status of RYGB was esteemed as a risk factor for kidney stones^64^. After RYGB, decreased urine output, decreased urine citrate, and increased urine oxalate are all visible pathogenetic factors for nephrolithiasis, meanwhile, through animal experiments, it was observed that Oxalobacter formigines, a non-pathogenic gut commensal that could consume oxalate, was colonized in mice to be able to reduce the increase of urinary oxalate after RYGB^65^, which suggests that the effect of bariatric surgery on intestinal flora may be a factor in the pathogenesis of kidney stones.

Based on epidemiological statistics, studies have indicated that men do have a higher incidence of kidney stones than women, by a ratio of about 2-3: 1^11,66^. Gender differences in nephrolithiasis incidence were usually attributed to hormonal differences between men and women, despite the exact mechanisms are still unclear. The existing literatures have reported that the promoting effect of androgen and the inhibiting effect of estrogen in urinary oxalate excretion^66^, but combined with our results, we could conclude that the promoting effect of androgen on kidney stones was more dominant than the inhibiting effect of estrogen. In addition, several studies have updated the role of androgen receptor (AR) signaling in nephrolithiasis formation, for example, upregulation of liver glycolate oxidase and renal epithelial nicotinamide adenine dinucleotide phosphate oxidase (NAPDH) to increase oxalate biosynthesis^67,68^, and inhibition of macrophage recruitment and its ability to phagocytose crystals^69^. Therefore, targeted therapy for AR can theoretically be used as a viable therapeutic intervention for kidney stones. In fact, dimethyl curcumin (ASC‑J9), a kind of AR degradation enhancer, has been reported to restrain oxalate crystal formation by controlling the kidney tubular epithelial cell injury and oxalate biosynthesis of rats^69^. Since the current progress is still in the research stage, the task of developing therapeutic drugs in this direction is still onerous.

From our results, higher temperatures, polycystic kidney disease, inflammatory bowel disease, cadmium exposure and pulp stones were believed to increase the risk of kidney stones, but according to the GREAD score, the evaluation results for these risk factors are not yet robust, indicating that further exploration and research are needed to help identify targeted treatment measures for possible factors that may lead to nephrolithiasis.

To sum up, the extensive and complex pathogenesis of kidney stones were observed by us, which did not only affect kidney singularly to result in the formation of stones. According to our article, various factors tended to affect the circulatory metabolism of the whole body, especially the metabolic association between liver, intestine and kidney, which suggests that kidney stones should be regarded as the consequence of systemic metabolic diseases, rather than ordinary kidney diseases.

The major strength of our study is that we systematically and thoroughly summarized and presented evidence of the associations between exposures and nephrolithiasis and then applied well-defined criteria to assess the credibility of the included studies. However, our study also had several limitations. Firstly, important exposures that were not reported in meta-analyses may be overlooked because umbrella reviews focus on existing meta-analyses. Secondly, outdated meta-analyses might provide incomplete conclusions with less power if a high-quality original study existed. Thirdly, we were unable to investigate the nonlinear causal associations between exposures and nephrolithiasis even if they did exist. More researches are needed to assess the causality in the future.

## Conclusion

In summary, our study demonstrates the suggestive causal (central obesity, T2D, gout, dietary sodium, fructose intake and higher temperatures) risk factors of nephrolithiasis. We also demonstrate the suggestive causal (coffee/alcohol/beer intake, dietary calcium and DASH-diet) protective factors of nephrolithiasis. It will be helpful to judge the relative priority of exposures associated with nephrolithiasis for future study and prevention of this disease. However, updated meta-analyses based on extensive prospective cohort studies for most risk factors are still urgently needed in the future.

## Ethics committee approval

No ethics committee approval was conducted because this study did not involve an animal and human experiments or data containing individual information.

## Consent

No ethics committee approval was conducted because this study did not involve an animal and human experiments or data containing individual information.

## Source of funding

This study was supported by the Foundation of Science & Technology Department of Sichuan Province [2022YFS0304, 2023YFS0029], the Post-Doctor Research Project, West China Hospital of Sichuan University[ 2020HXBH016], the National Natural Science Foundation of China [82200851] and the Natural Science Foundation of Sichuan Province of China [2023NSFSC1533].

## Author contribution

The authors’ responsibilities were as follows: C.C., M.Y., J.Z., W.J., L.X., L.H., W.K., J.X. for concept and design; C.C., M.Y. for the data acquisition; J.Z., M.Y., X.L. for statistical analysis and interpretation of data; C.C. for drafting of the manuscript; W.J., L.H., W.K., J.X. for critical revision of the manuscript. All authors interpreted data, revised the document, and read and approved the final manuscript.

## Conflicts of interest disclosure

None.

## Research registration unique identifying number (UIN)

The umbrella review was registered with Research Registry (researchregistry10240).

## Guarantor

Xi Jin.

## Data availability statement

All data generated or analyzed during this study are included in this article. The data are available from the corresponding author upon reasonable request.

## Provenance and peer review

Not commissioned, externally peer-reviewed.

## Supplementary Material
