# Supplementary material for: Risk factors for nephrolithiasis formation: an umbrella review
Source: Int J Surg. 2024 May 29;110(9):5733–44. doi: 10.1097/JS9.0000000000001719 (PMC11392093; doi:10.1097/JS9.0000000000001719)
Supplement: Supplementary file 1 [file js9-110-5733-s001.docx]

**Umbrella Review Provides Mutually Complementary Insight for Risk Factors of Nephrolithiasis.**

Supplementary Materials 1. Searchable details for inclusion in the Pubmed database.

Supplementary Materials 2. Specific criteria for evaluating credibility.

Supplementary Table S1. Detailed evaluation of the methodological quality of the included systematic reviews and meta-analyses with AMSTAR2.

Supplementary Table S2. Study selection for umbrella review based on AMSTAR2.0 tools.

Supplementary Table S3. Grading of Recommendations Assessment, Development and Evaluation assessment of each association: meta-analysis study.

**Supplementary Materials 1. Searchable details for inclusion in the Pubmed database.**

((((((((((((((((((((((((((((((((((((((((((((((((((((((nephrolithiasis[Title/Abstract]) OR (calculosis, kidney[Title/Abstract])) OR (calculus, kidney[Title/Abstract])) OR (familial nephrolithiasis[Title/Abstract])) OR (kidney calculi[Title/Abstract])) OR (kidney calculosis [Title/Abstract])) OR (kidney calculus[Title/Abstract])) OR (kidney calix stone[Title/Abstract])) OR (kidney calyx stone[Title/Abstract])) OR (kidney lithiasis[Title/Abstract])) OR (kidney pelvis stone[Title/Abstract])) OR (kidney stone[Title/Abstract])) OR (kidney stone passage[Title/Abstract])) OR (kidney stone, pelvis[Title/Abstract])) OR (kidney stones[Title/Abstract])) OR (nephrolith[Title/Abstract])) OR (nephrolith passage[Title/Abstract])) OR (nephrolithiasis[Title/Abstract])) OR (renal calculogenesis[Title/Abstract])) OR (renal calculosis[Title/Abstract])) OR (renal calculus[Title/Abstract])) OR (renal lithiasis[Title/Abstract])) OR (renal pelvis stone[Title/Abstract])) OR (renal stone[Title/Abstract])) OR (renolithiasis[Title/Abstract])) OR (stone, kidney[Title/Abstract])) OR (urolithiasis[Title/Abstract])) OR (coral stone[Title/Abstract])) OR (stone, urinary tract[Title/Abstract])) OR (stone, urine[Title/Abstract])) OR (urinary calculi[Title/Abstract])) OR (urinary calculogenesis[Title/Abstract])) OR (urinary calculoses[Title/Abstract])) OR (urinary calculosis[Title/Abstract])) OR (urinary calculus[Title/Abstract])) OR (urinary lithiasis[Title/Abstract])) OR (urinary stone[Title/Abstract])) OR (urinary tract calculi[Title/Abstract])) OR (urinary tract calculosis[Title/Abstract])) OR (urinary tract calculus[Title/Abstract])) OR (urinary tract lithiasis[Title/Abstract])) OR (urinary tract stone[Title/Abstract])) OR (urine calculi[Title/Abstract])) OR (urine calculus[Title/Abstract])) OR (urine lithiasis[Title/Abstract])) OR (urine stone[Title/Abstract])) OR (uro-lithiasis[Title/Abstract])) OR (urocalculi[Title/Abstract])) OR (urocalculogenesis[Title/Abstract])) OR (urocalculosis[Title/Abstract])) OR (urolith[Title/Abstract])) OR (urolithogenesis[Title/Abstract])) OR (urologic calculi[Title/Abstract])) OR (urological calculi[Title/Abstract])) OR (urolyt[Title/Abstract]) AND meta-analysis.

**Supplementary Materials 2. Specific criteria for evaluating credibility.**

Class I (Convincing):

(1) Statistical significance under random effects: P < 10E-06;

(2) More than 1000 cases;

(3) Statistical significance in the largest component study: P < 0.05;

(4) 95% prediction interval excluded the null;

(5) No large heterogeneity (I^2^ < 50%), and no evidence of small study effects and of excess significance bias.

Class II (Highly suggestive):

(1) Statistical significance under random effects: P < 10E-06;

(2) More than 1000 cases;

(3) Statistical significance in the largest component study: P < 0.05;

Class III (Suggestive):

(1) Statistical significance under random effects: P < 0.001;

(2) More than 1000 cases;

Class IV (Weak):

(1) Statistical significance under random effects: P < 0.05;

Not significant:

(1) Statistical significance under random effects: P > 0.05.

**Supplementary Table S1. Detailed evaluation of the methodological quality of the included systematic reviews and meta-analyses with AMSTAR2.**

| Author, year | Question 1 | Question 2 | Question 3 | Question 4 | Question 5 | Question 6 | Question 7 | Question 8 | Question 9 | Question 10 | Question 11 | Question 12 | Question 13 | Question 14 | Question 15 | Question 16 | Quality |
| --- | --- | --- | --- | --- | --- | --- | --- | --- | --- | --- | --- | --- | --- | --- | --- | --- | --- |
| Bing-Biao Lin (2020) [3] | Yes | No | Yes | Yes | Yes | No | Partial Yes | Partial Yes | Yes | No | Yes | No | Yes | Yes | Yes | Yes | Low |
| Dagfinn Aune (2018) [4] | Yes | No | Yes | Yes | No | Yes | Partial Yes | Yes | Yes | No | Yes | Yes | Yes | Yes | Yes | Yes | Low |
| Bing-Biao Lin (2020) [5] | Yes | No | No | Yes | No | No | Partial Yes | Yes | No | No | Yes | No | Yes | Yes | Yes | Yes | Critially Low |
| Huseyin Besiroglu (2018) [6] | Yes | No | Yes | Partial Yes | No | No | Partial Yes | Partial Yes | No | No | Yes | Yes | Yes | Yes | Yes | Yes | Low |
| Shaoyou Qin (2018) [7] | Yes | No | No | Yes | No | No | Partial Yes | Partial Yes | No | No | Yes | No | No | Yes | Yes | Yes | Critially Low |
| Karn Wijarnpreecha (2018) [8] | Yes | No | Yes | Yes | Yes | Yes | Partial Yes | Yes | Partial Yes | No | Yes | No | Yes | Yes | Yes | Yes | Low |
| Xiao Wang (2014) [9] | Yes | No | Yes | No | No | Yes | Partial Yes | Partial Yes | Yes | No | Yes | Yes | Yes | Yes | Yes | Yes | Critially Low |
| Chang Xu (2015) [10] | Yes | No | Yes | Yes | No | Yes | Partial Yes | Yes | Yes | No | Yes | No | Yes | Yes | Yes | Yes | Low |
| Shuai Wang (2014) [11] | Yes | No | Yes | Partial Yes | Yes | Yes | Partial Yes | Partial Yes | Yes | No | Yes | Yes | Yes | Yes | Yes | Yes | Low |
| Lu Hao Liu (2015) [12] | Yes | No | Yes | Partial Yes | No | Yes | Partial Yes | Partial Yes | No | No | Yes | Yes | Yes | Yes | Yes | Yes | Critially Low |
| Mahboube Ganji-Arjenaki (2017) [13] | Yes | No | No | No | No | No | No | No | No | No | No | No | No | No | No | Yes | Critially Low |
| Robert Geraghty (2019) [14] | Yes | Yes | Yes | Yes | Yes | No | Partial Yes | Yes | Yes | No | Yes | Yes | Yes | Yes | Yes | Yes | Moderate |
| Charat Thongprayoon (2016) [15] | Yes | No | Yes | No | Yes | Yes | Partial Yes | Yes | No | No | Yes | No | Yes | Yes | No | Yes | Critially Low |
| Sikarin Upala (2016) [16] | Yes | Yes | Yes | Yes | Yes | Yes | Yes | Yes | Yes | No | No | No | Yes | Yes | No | Yes | Critially Low |
| Juan Yu (2017) [17] | Yes | No | Yes | Partial Yes | No | No | Partial Yes | Partial Yes | No | No | Yes | Yes | Yes | Yes | Yes | Yes | Critially Low |
| Marilisa Carneiro Leao Gabardo (2019) [18] | Yes | Yes | Yes | Yes | Yes | No | Yes | Yes | No | Yes | No | No | Yes | Yes | No | Yes | Critially Low |
| Wisit Cheungpasitporn (2016) [19] | Yes | No | Yes | Partial Yes | Yes | Yes | Partial Yes | Yes | Yes | Yes | No | Yes | Yes | Yes | Yes | Yes | Low |
| Matthew J Roughley (2015) [20] | Yes | No | Yes | Yes | Yes | Yes | Partial Yes | Yes | Yes | No | Yes | Yes | Yes | Yes | Yes | Yes | Low |
| Zhen-Lang Guo (2017) [21] | Yes | Yes | Yes | Yes | Yes | Yes | Partial Yes | Partial Yes | Yes | No | Yes | Yes | Yes | Yes | No | Yes | Low |
| Gianpaolo Perletti (2020) [22] | Yes | No | Yes | Yes | No | Yes | No | No | Yes | No | No | No | Yes | Yes | Yes | Yes | Critially Low |
| Domenico Rendina (2014) [23] | Yes | No | No | Yes | Yes | Yes | No | No | No | No | Yes | No | Yes | No | Yes | Yes | Critially Low |
| Zarintaj Malihi (2016) [24] | No | No | Yes | Partial Yes | No | Yes | Partial Yes | Yes | Yes | No | Yes | Yes | Yes | Yes | Yes | No | Critially Low |
| Vinusha Kalatharan (2020) [25] | Yes | No | No | Yes | Yes | Yes | Partial Yes | Yes | Yes | No | Yes | No | Yes | Yes | No | Yes | Critially Low |
| Woo-Seok Lee (2019) [26] | Yes | No | No | Partial Yes | Yes | Yes | Partial Yes | Yes | Yes | No | Yes | Yes | Yes | Yes | Yes | Yes | Low quality |

**Supplementary Table S2. Study selection for umbrella review based on AMSTAR2.0 tools.**

| **Study** | **Number of included cohorts** | **Estimate metrics** | **Reported summary estimate** | **Amstar score** | **Whether included in Umbrella review** | **Unit used in included meta-analysis for continuous factors** |
| --- | --- | --- | --- | --- | --- | --- |
| ***Metabolic factors*** | | | | | | |
| **Waist circumference** | | | | | | |
| Aune (2018) [4] | 5 cohorts | RR | 1.16 (1.12, 1.19) | Critically Low-quality review | Yes | Per-10cm increase (dose-response) |
| **BMI** | | | | | |  |
| Lin (2020) [3] | 16 cohorts | RR | 1.39 (1.27, 1.52) | Low-quality review | No | / |
| Aune (2018) [4] | 8 cohorts | RR | 1.21 (1.12, 1.30) | Low-quality review | Yes (Despite the similar quality, this study provides a dose-response analysis) | Per 5-unit increase (dose-response) |
| **T2D** | | | | | | |
| Lin (2020) [5] | 12 cohorts | RR | 1.18 (1.07, 1.29) | Critically Low-quality review | Yes | / |
| Aune (2018) [4] | 10 cohorts | RR | 1.16 (1.03, 1.31) | Critically Low-quality review | No | / |
| Liu (2015) [12] | 7 cohorts | RR | 1.24 (1.14, 1.35) | Critically Low-quality review | No | / |
| **Gout** | | | | | | |
| Roughley (2015) [20] | 3 cohorts | OR | 1.77 (1.43, 2.19) | Low-quality review | Yes | / |
| **NAFLD** | | | | | | |
| Qin (2018) [7] | 8 cohorts | OR | 1.73 (1.24, 2.40) | Critically Low-quality review | No | / |
| Wijarnpreecha (2018) [8] | 8 cohorts | OR | 1.81 (1.29, 2.56) | Low-quality review | Yes | / |
| **Metabolic syndrome** | | | | | | |
| Rendina (2014) [23] | 7 cohorts | OR | 1.29 (1.11, 1.51) | Critically Low-quality review | No | / |
| Geraghty (2019) [14] | 6 cohorts | OR | 1.35 (1.16, 1.55) | Moderate quality review | Yes | / |
| **HDL-C and triglycerides level** | | | | | | |
| Besiroglu (2018) [6] | 9 cohorts for HDL-C;  8 cohorts for triglycerides | OR | 1.17 (1.01, 1.36) for HDL-C;  1.29 (1.07, 1.54) for triglycerides | Low-quality review | Yes | HDL-C for men < 1.03 mmol/L (or < 40 mg/dL); HDL-C for women < 1.3 mmol/L (or < 50 mg/dL); Triglycerides ≥ 150 mg/dL or triglycerides > 1.7 mmol/L. |
| **Hypertension and gallstones** | | | | | | |
| Lin (2020) [5] | 13 cohorts for hypertension;  6 for gallstones | RR | 1.30 (1.11, 1.52) for hypertension;  1.46 (1.15, 1.85) for gallstones | Critically Low-quality review | Yes | / |
| **Impaired glucose tolerance** | | | | | | |
| Geraghty (2019) [14] | 4 Cohorts | OR | 1.26 (0.94, 1.58) | Moderate quality review | Yes | / |
| ***Fluid and beverages intake*** | | | | | | |
| **Soda** | | | | | | |
| Xu (2015) [10] | 4 cohorts | RR | 1.03 (0.90, 1.17) | Low-quality review | No | / |
| Lin (2020) [3] | 5 cohorts | RR | 1.38 (1.26, 1.56) | Low-quality review | Yes | Relative risk of kidney stones introduced by per bottle of soda (mentioned as soft drinks) (330ml);  Incidence rate ratio of kidney stones introduced in comparison between soda consumption ≥7 servings per week and < 4 serving per week;  Odds ratio of kidney stone risk introduced by soda consumption habit (Drinking soda compared with never drinking). |
| **Caffeine** | | | | | | |
| Lin (2020) [3] | 3 cohorts | HR | 0.71 (0.64, 0.79) | Low-quality review | Yes | Hazard ratio of kidney stones for 20% of the population with the highest intake of caffeine compared with the 20% of the population with the least intake. |
| **Fluid** | | | | | | |
| Cheungpasitporn (2016) [19] | 9 cohorts | RR | RCTs: 0.40 (0.20, 0.79)  NRIs: 0.49 (0.34, 0.71)  All: 0.47 (0.33, 0.67) | Low-quality review | Yes | Water/fluid intake > 1.8L/d-2.7L/d, or fluid intake to increase urine volume 1L/d-2L/d. |
| Lin (2020) [4] | 5 cohorts | RR | 0.90 (0.84, 0.97) | Low-quality review | No | / |
| Xu (2015) [10] | 6 cohorts | RR | 0.93 (0.87, 0.98) | Low-quality review | No | / |
| **Alcohol** | | | | | | |
| Wang (2014) [9] | 12 cohorts | OR | 0.68 (0.58, 0.81) | Critically Low-quality review | No | / |
| Lin (2020) [3] | 7 cohorts | RR | 0.69 (0.56, 0.85) | Low-quality review | Yes | Relative risk (or hazard ratio) of kidney stones for 25% of the population with the highest intake of alcohol compared with the 25% of the population with the least intake;  Odds ratio of kidney stone risk for people with frequently alcohol consumption (3 drinks/ month) compared with people without alcohol consumption;  Hazard ratio of kidney stone risk for population with alcohol consumption more than 16g/day (or ≥46g/day, or ≥4 glasses/day) compared with less than 1g/day (or never). |
| **Beer** | | | | | | |
| Lin (2020) [3] | 5 cohorts | RR | 0.60 (0.49, 0.74) | Low-quality review | Yes | Odds ratio of kidney stones introduced by beer consumption habit (Drinking beer compared with never drinking) or comparison between population have beer >300 mL/day and never;  Relative risk of kidney stones introduced by per bottle (330ml) of beer;  Relative risk of kidney stones for population have beer more than 1 serving/day compared with less than 1 serving/week. |
| **Coffee** | | | | | | |
| Lin (2020) [3] | 6 cohorts | RR | 0.82 (0.70, 0.97) | Low-quality review | No | / |
| Xu (2015) [10] | 5 cohorts | RR | 0.88 (0.76, 1.00) | Low-quality review | No | / |
| Wang (2014) [11] | 10 cohorts | OR | 0.70 (0.60, 0.82) | Low-quality review | Yes | Ever vs. never or ≥4-6 cups/day vs. <1 cup/month or ≥1 time/week vs. <1 time/month. |
| **Tea** | | | | | |  |
| Lin (2020) [3] | 8 cohorts | RR | 0.88 (0.79, 0.97) | Low-quality review | Yes | Odds ratio of kidney stone risk introduced by tea consumption habit (Drinking beer compared with never drinking) or comparison between population have tea ≥1 time/day and <1 times/month;  Relative risk of kidney stones introduced by per cup of tea (170ml) or relative risk of kidney stones for population have beer more than 1 serving/day compared with less than 1 serving/week;  Hazard ratio of kidney stone risk for population with tea consumption ≥5cups/day compared with never. Hazard ratio of kidney stone risk for population with tea consumption ≥150g dried tea leaf per month compared with population without tea consumption. |
| Xu (2015) [10] | 7 cohorts | RR | 1.06 (0.94, 1.20) | Critically Low-quality review | No | / |
|  | | | | | | |
|  |  |  |  |  |  |  |
| **Milk** | | | | | | |
| Xu (2015) [10] | 5 cohorts | RR | 0.95 (0.86, 1.05) | Low-quality review | Yes | 1-2 serving per day |
| **Juice** | | | | | | |
| Xu (2015) [10] | 3 cohorts | RR | 1.00 (0.92, 1.09) | Low-quality review | Yes | 1-2 serving per day |
| ***Vitamin and calcium intake*** | | | | | | |
| **Calcium supplement** | | | | | | |
| Lin (2020) [3] | 2 cohorts | RR | 1.16 (1.00, 1.35) | Low-quality review | Yes | Relative risk of kidney stones introduced by comparison between the population with calcium supplement ≥501mg/day and the population without calcium supplement. |
| **Dietary calcium intake** |  |  |  |  |  |  |
| Lin (2020) [3] | 6 cohorts | RR | 0.83 (0.76, 0.90) | Low-quality review | Yes | Relative risk of kidney stones introduced by comparison between the 25% of the population with the highest intake of dietary calcium intake and the 25% of the population with the least dietary calcium intake;  Odds ratio of kidney stones introduced by comparison between the 20% of the population with the highest intake of dietary calcium intake and the 20% of the population with the least dietary calcium intake; |
| **Total vitamin D** | | | | | | |
| Lin (2020) [3] | 4 cohorts | RR | 1.07 (0.93, 1.23) | Low-quality review | Yes | Relative risk of kidney stones introduced by comparison between the 25% of the population with the highest intake of total vitamin D intake and the 25% of the population with the least total vitamin D intake;  Hazard ratio of kidney stones introduced by comparison between the population with total vitamin D intake ≥1000IU/day and the population with total vitamin D intake<100IU/day. |
| **Vitamin D supplement** | | | | | | |
| Lin (2020) [3] | 5 cohorts | RR | 1.22 (1.01, 1.49) | Low-quality review | Yes | Odds ratio of kidney stones introduced by comparison between the population with vitamin D supplement and the population without vitamin D supplement;  Hazard ratio of kidney stones introduced by comparison between the population with total vitamin D supplement ≥1000IU/day and the population without supplement. |
| **Total vitamin C** | | | | | | |
| Lin (2020) [3] | 3 cohorts | RR | 1.15 (0.90, 1.48) | Low-quality review | Yes | Relative risk of kidney stones introduced by comparison between the 25% of the population with the highest intake of total vitamin C and the 25% of the population with the least total vitamin C intake. |
| **Vitamin C supplement** | | | | | | |
| Lin (2020) [3] | 8 cohorts | RR | 1.10 (0.95, 1.26) | Low-quality review | Yes | Relative risk of kidney stones introduced by comparison between the population with vitamin C supplement and the population without vitamin C supplement;  Hazard ratio of kidney stones introduced by comparison between the population with vitamin C supplement ≥1000mg/day and the population without vitamin C supplement. |
| Malihi (2016) [24] | 9 cohorts | RR | 0.66 (0.41, 1.09) | Critically Low-quality review | No | / |
| **Total vitamin B6** | | | | | | |
| Lin (2020) [3] | 4 cohorts | RR | 1.01 (0.91, 1.12) | Low-quality review | Yes | Relative risk of kidney stones introduced by comparison between the 25% of the population with the highest intake of total vitamin C and the 25% of the population with the least total vitamin C intake. |
| ***Dietary intake*** | | | | | | |
| **Dietary sodium** | | | | | | |
| Lin (2020) [3] | 4 cohorts | RR | 1.38 (1.21, 1.56) | Low-quality review | Yes | Relative risk of kidney stones introduced by comparison between the 20% (33%) of the population with the highest intake of dietary sodium intake and the 20% (33%) of the population with the least dietary sodium intake;  Hazar ratio of kidney stones introduced by comparison between the population always add salt to food and the 3 population never/rarely add salt to food. |
| **Fructose** | | | | | | |
| Lin (2020) [3] | 3 cohorts | RR | 1.33 (1.19, 1.49) | Low-quality review | Yes | Relative risk of kidney stones introduced by comparison between the 20% of the population with the highest intake of total fructose and the 20% of the population with the least fructose intake. |
| **Meat (regardless of meat type)** | | | | | | |
| Lin (2020) [3] | 5 cohorts | RR | 1.24 (1.12, 1.39) | Low-quality review | Yes | Odds ratio of kidney stones introduced by comparison between the 33% of the population with the highest intake of meat and the 33% of the population with the least meat intake;  Hazard ratio of kidney stones introduced by comparison between the 20% of the population with the highest intake of meat and the 20% of the population with the least meat intake;  Hazard ratio of kidney stones introduced by comparison between the population with ≥9.25 servings/week and the population with 0-3 servings/week. |
| **Spinach** | | | | | | |
| Lin (2020) [3] | 3 cohorts | RR | 1.21 (1.01, 1.44) | Low-quality review | Yes | Relative risk of kidney stones introduced by comparison between population who have ≥8 servings/month of spinach and population who have spinach fewer than 1 serving. |
| **Fiber intake** |  |  |  |  |  |  |
| Lin (2020) [3] | 3 cohorts | RR | 0.71 (0.64, 0.79) | Low-quality review | Yes | Hazard ratio of kidney stones introduced by comparison between the 20% (or 33%) of the population with the highest intake of fiber and the 20% (or 33%) of the population with the least intake. |
| **DASH-style diet** | | | | | | |
| Lin (2020) [3] | 3 cohorts | RR | 0.69 (0.64, 0.75) | Low-quality review | Yes | / |
| **Dietary potassium** | | | | | | |
| Lin (2020) [3] | 4 cohorts | RR | 0.59 (0.46, 0.75) | Low-quality review | Yes | Relative risk of kidney stones introduced by comparison between the 20% (33%) of the population with the highest intake of dietary potassium intake and the 20% (33%) of the population with the least dietary potassium intake;  Hazar ratio of kidney stones introduced by comparison between the 33% of the population with the highest intake of dietary potassium intake and the 33% of the population with the least dietary potassium intake. |
| **Dietary magnesium** | | | | | | |
| Lin (2020) [3] | 3 cohorts | RR | 0.66 (0.55, 0.79) | Low-quality review | Yes | Relative risk of kidney stones introduced by comparison between the 25% (20%) of the population with the highest intake of dietary magnesium intake and the 25% (20%) of the population with the least dietary magnesium intake;  Hazar ratio of kidney stones introduced by comparison between the 33% of the population with the highest intake of dietary magnesium intake and the 33% of the population with the least dietary magnesium intake. |
| **Fruit** | | | | | | |
| Lin (2020) [3] | 6 cohorts | RR | 0.79 (0.71, 0.89) | Low-quality review | Yes | Odds ratio of kidney stone risk introduced by comparison between population have fruit ≥1 time/day and <1 times/month;  Hazard ratio of kidney stones introduced by comparison between the 20 % (33%) of the population with the highest intake of fruit and the 20% (33%) of the population with the least fruit intake;  Hazard ratio of kidney stones introduced by comparison between the population have fruit ≥5 servings/day and population have fruit 0-1 servings/day. |
| **Vegetable** | | | | | | |
| Lin (2020) [3] | 4 cohorts | RR | 0.84 (0.75, 0.94) | Low-quality review | Yes | Hazard ratio of kidney stones introduced by comparison between the 20 % (33%) of the population with the highest intake of vegetables and the 20% (33%) of the population with the least vegetable intake; Hazard ratio introduced by comparison between population have vegetable ≥ 7 tablespoons/day and population have vegetable 0-2 tablespoons/day. |
| **Energy** | | | | | | |
| Lin (2020) [3] | 5 cohorts | RR | 1.12 (0.99, 1.27) | Low-quality review | Yes | Hazard ratio of kidney stones introduced by comparison between the 33% of the population with the highest intake of energy and the 33% of the population with the least energy intake. (1 study just mentioned highest vs. lowest, no detailed threshold value offered);  Hazard ratio introduced by comparison between population with energy intake ≥ 2500 kcal/day and population with energy intake <1800 kcal/d/day. |
| Perletti (2020) [22] | 4 cohorts | OR | 1.95 (1.87, 5.56) | Critically Low-quality review | No | / |
| ***Other factors*** | | | | | | |
| **Temperatures** | | | | | | |
| Lee (2019) [26] | 8 cohorts | RR | 1.30 (1.20, 1.41) | Low-quality | Yes | Higher mean temperature vs. lower mean temperature, or heat wave vs. normal climate. |
| **Polycystic kidney disease** | | | | | | |
| Kalatharan (2020) [25] | 6 cohorts | RR | 1.85 (1.29, 2.64) | Critically Low-quality review | Yes | / |
| Xu (2015) [10] | 7 cohorts | RR | 0.80 (0.63, 1.01) | Low-quality review | No | / |
| **Inflammatory bowel disease** | | | | | | |
| Ganji-Arjenaki (2017) [13] | 5 cohorts | OR | 3.86 (1.14, 13.03) | Critically Low-quality review | Yes* | / |
| **Cadmium exposure** | | | | | | |
| Guo (2017) [21] | 6 cohorts | OR | 1.32 (1.08, 1.62) | Low-quality review | Yes | / |
| **Pulp Stones** | | | | | | |
| Gabardo (2019) [18] | 2 cohorts | OR | 1.97 (1.21, 3.18) | Critically Low-quality review | Yes | / |
| **Physical activity** | | | | | | |
| Aune (2018) [4] | 4 cohorts | RR | 0.91 (0.73, 1.12) | Critically Low-quality review | Yes | Per 20 MET-hrs (dose-response) |
| Lin (2020) [3] | 7 cohorts | RR | 0.91 (0.81, 1.02) | Low-quality review | No | / |
| **Bariatric surgery** | | | | | | |
| Thongprayoon (2016) [15] | 4 cohorts | RR | 1.22 (0.63, 2.35) | Critically Low-quality review | Yes | / |
| Upala (2016) [16] | 5 cohorts | RR | 1.79 (1.54, 2.10) | Critically Low-quality review | No | / |
| **Postmenopausal hormone** | | | | | | |
| Yu (2017) [17] | 7 cohorts | RR | 0.91 (0.72, 1.14) | Critically Low-quality review | Yes | / |

BMI, body mass index; T2D, type 2 diabetes; NAFLD, non-alcoholic fatty liver disease; HDL-C, high-density lipoprotein cholesterol; 2hGlu, 2-h glucose after an oral glucose challenge; 25(OH)D, 25-Hydroxyvitamin D; MET, metabolic equivalent of task.

**Supplementary Table S3. Grading of Recommendations Assessment, Development and Evaluation assessment of each association.**

|  |  | | Downgrade |  | |  | |  |  | | | Upgrade |  |  |
| --- | --- | --- | --- | --- | --- | --- | --- | --- | --- | --- | --- | --- | --- | --- |
| Exposure | Study design | | Risk of Bias | Inconsistency | | Indirectness | | Imprecision | Publication bias | | | Large magnitude of effect | Dose-response | GRADE |
| ***Metabolic factors*** | | | | | | | | | | | | | | |
| Waist circumference | High | | Not serious | Not serious | | Not serious | | Not serious | Not serious | | | No | Yes | High |
| BMI | Low | | Not serious | Serious | | Not serious | | Not serious | Not serious | | | No | Yes | Moderate |
| T2D | Low | | Not serious | Serious | | Not serious | | Not serious | Not serious | | | No | No | Very low |
| Gout | Low | | Not serious | Not serious | | Not serious | | Not serious | Not serious | | | No | No | Low |
| NAFLD | Low | | Not serious | Very Serious | | Not serious | | Not serious | Not serious | | | No | No | Very low |
| Metabolic syndrome | Low | | Not serious | Very serious | | Not serious | | Not serious | Not serious | | | No | No | Very low |
| HDL-C (Decreased) | Low | | Serious | Very Serious | | Not serious | | Not serious | Not serious | | | No | No | Very low |
| Triglycerides | Low | | Serious | Very Serious | | Not serious | | Not serious | Serious | | | No | No | Very low |
| Hypertension | High | | Not serious | Very Serious | | Not serious | | Not serious | Serious | | | No | No | Very low |
| Gallstones | High | | Not serious | Very Serious | | Not serious | | Not serious | Not serious | | | No | No | Low |
| Impaired glucose tolerance | Low | | Not serious | Very serious | | Not serious | | Not serious | Not serious | | | No | No | Very low |
| ***Fluid and beverages intake*** | | | | | | | | | | | | | | |
| Soda | Low | | Serious | Not serious | | Not serious | | Not serious | Not serious | | | No | No | Very low |
| Caffeine | High | | Not serious | Not serious | | | Not serious | Not serious | | | Not serious | No | No | High |
| Fluid | Low | | Not serious | Very serious | | Not serious | | Not serious | Not serious | | | No | No | Very low |
| Alcohol | Low | | Not serious | Serious | | Not serious | | Not serious | Not serious | | | No | No | Very low |
| Beer | Low | | Serious | Not serious | | Not serious | | Not serious | Not serious | | | No | No | Very low |
| Tea | Low | | Not serious | Serious | | Not serious | | Not serious | Not serious | | | No | No | Very low |
| Coffee | Low | | Serious | Not serious | | Not serious | | Serious | Not serious | | | No | No | Very low |
| Milk | Low | | Not serious | Serious | | Not serious | | Not serious | Not serious | | | No | Yes | Low |
| Juice | Low | | Serious | Not serious | | Not serious | | Not serious | Not serious | | | No | Yes | Low |
| ***Vitamin and calcium intake*** | | | | | | | | | | | | | | |
| Calcium Supplementation | High | | Not serious | Not serious | | Not serious | | serious | Serious | | | No | No | Low |
| Dietary calcium | High | | Not serious | Not serious | | Not serious | | Not serious | Not serious | | | No | No | High |
| Total vitamin D | High | | Not serious | Not serious | | Not serious | | Not serious | Not serious | | | No | No | High |
| Vitamin D Supplement | Low | | Not serious | Not serious | | Not serious | | Not serious | Serious | | | No | No | Very low |
| Total vitamin C intake | High | | Not serious | Very serious | | Not serious | | Serious | Not serious | | | No | No | Very low |
| Vitamin C Supplement | Low | | Not serious | Serious | | Not serious | | Serious | Not serious | | | No | No | Very low |
| Total vitamin B6 intake | High | | Not serious | Not serious | | Not serious | | Not serious | Serious | | | No | No | Moderate |
| ***Dietary intake*** | | | | | | | | | | | | | | |
| Dietary sodium intake | High | | Not serious | Not serious | | Not serious | | Not serious | Not serious | | | No | No | High |
| Fructose | High | | Not serious | Not serious | | Not serious | | Not serious | Not serious | | | No | No | High |
| Meat | Low | | Not serious | Not serious | | Not serious | | Not serious | Not serious | | | No | No | Low |
| Spinach intake | High | | Not serious | Serious | | Not serious | | Not serious | Not serious | | | No | No | Moderate |
| Fiber | High | | Not serious | Not serious | | Not serious | | Not serious | Not serious | | | No | No | High |
| DASH-style diet | High | | Not serious | Not serious | | Not serious | | Not serious | Not serious | | | No | No | High |
| Dietary potassium | High | | Not serious | Very serious | | Not serious | | Not serious | Not serious | | | No | No | Low |
| Dietary magnesium | High | | Not serious | Not serious | | Not serious | | Not serious | Serious | | | No | No | Moderate |
| Fruit | Low | | Not serious | Not serious | | Not serious | | Not serious | Not serious | | | No | No | Low |
| Vegetable | High | | Not serious | Not serious | | Not serious | | Not serious | Not serious | | | No | No | High |
| Energy | | High | Not serious | | Not serious | | Not serious | Not serious | | | Serious | No | No | Moderate |
| ***Other factors*** | | | | | | | | | | | | | | |
| High temperatures | Low | | Not serious | Not serious | | Serious | | Not serious | Not serious | | | No | No | Very low |
|  |  | |  |  | |  | |  |  | | |  |  |  |
| Polycystic kidney disease | Low | | Serious | Not serious | | Not serious | | Not serious | Not serious | | | No | No | Very low |
| Inflammatory bowel disease | Low | | Serious | Very Serious | | Not serious | | Not serious | Not serious | | | Yes | No | Very low |
| Cadmium exposure | Low | | Serious | Serious | | Not serious | | Serious | Serious | | | No | No | Very low |
| Pulp Stones | Low | | Not serious | Not serious | | Not serious | | Serious | | Serious | | No | No | Very low |
| Physical activity | High | | Not serious | Serious | | Not serious | | Serious | Not serious | | | No | Yes | High |
| Bariatric surgery | Low | | Not serious | Very serious | | Not serious | | Serious | Not serious | | | No | No | Very low |
| Postmenopausal hormone | Low | | Serious | Serious | | Not serious | | Serious | Serious | | | No | No | Very low |

BMI, body mass index; T2D, type 2 diabetes; NAFLD, non-alcoholic fatty liver disease; HDL-C, high-density lipoprotein cholesterol; DASH-diet, dietary approaches to stop hypertension-diet.

Grading rationale: Study design: Cohort study, high; case-control or cross-sectional study, low. If the meta-analysis includes multiple types of original research, the lowest-level research is used for grading.

1.Risk of Bias: We assigned ‘serious’ when studies with a Newcastle-Ottawa scale score < 7 comprised a large (30%) proportion； ‘very serious’ when studies with a Newcastle-Ottawa scale score < 7 comprised a most (50%) proportion

2.Inconsistency: We assigned ‘inconsistency’ by accounting heterogeneity measured by the I2 statistic, variability in point estimates, and extent of overlap in confidence intervals.

3.Indirectness: We assigned ‘indirectness’ when the outcome was measured by surrogate indicators (such as renal colic) or was derived from study populations that differed from those of interest.

4.Publication bias: We assigned ‘detected’ when substantial asymmetry was observed in the funnel plot, or when the p value was <0.10 in Egger’s test.

5.Imprecision: We assigned ‘imprecision’ when the sample size was too small (<1000 cases) or the confidential interval (CI) was too large.

6.Large magnitude of effect: We assigned ‘large magnitude’ when the relative risk or equivalent was >2 (or <0.5).

7.Dose-response association: We assigned ‘dose-response’ when the effect size showed statistically significant increment per unit increases in adiposity indices.

When the relevant information is not provided by the original research, there will be relevant degrade items by default, but no upgrade items applied.

References

[1] Leong A, Cole JB, Brenner LN, Meigs JB, Florez JC, Mercader JM. Cardiometabolic risk factors for COVID-19 susceptibility and severity: A Mendelian randomization analysis. PLoS Med. 2021;18:e1003553.

[2] Burgess S, Thompson SG, Collaboration CCG. Avoiding bias from weak instruments in Mendelian randomization studies. Int J Epidemiol. 2011;40:755-64.

[3] Lin BB, Lin ME, Huang RH, Hong YK, Lin BL, He XJ. Dietary and lifestyle factors for primary prevention of nephrolithiasis: a systematic review and meta-analysis. BMC Nephrol. 2020;21:267.

[4] Aune D, Mahamat-Saleh Y, Norat T, Riboli E. Body fatness, diabetes, physical activity and risk of kidney stones: a systematic review and meta-analysis of cohort studies. Eur J Epidemiol. 2018;33:1033-47.

[5] Lin BB, Huang RH, Lin BL, Hong YK, Lin ME, He XJ. Associations between nephrolithiasis and diabetes mellitus, hypertension and gallstones: A meta-analysis of cohort studies. Nephrology (Carlton). 2020;25:691-9.

[6] Besiroglu H, Ozbek E. Association between blood lipid profile and urolithiasis: A systematic review and meta-analysis of observational studies. Int J Urol. 2019;26:7-17.

[7] Qin S, Wang S, Wang X, Wang J. Non-alcoholic fatty liver disease and the risk of urolithiasis: A systematic review and meta-analysis. Medicine (Baltimore). 2018;97:e12092.

[8] Wijarnpreecha K, Lou S, Panjawatanan P, Sanguankeo A, Pungpapong S, Lukens FJ, et al. Nonalcoholic Fatty Liver Disease and Urolithiasis. A Systematic Review and Meta-Analysis. J Gastrointestin Liver Dis. 2018;27:427-32.

[9] Wang X, Xu X, Wu J, Zhu Y, Lin Y, Zheng X, et al. Systematic review and meta-analysis of the effect of alcohol intake on the risk of urolithiasis including dose-response relationship. Urol Int. 2015;94:194-204.

[10] Xu C, Zhang C, Wang XL, Liu TZ, Zeng XT, Li S, et al. Self-Fluid Management in Prevention of Kidney Stones: A PRISMA-Compliant Systematic Review and Dose-Response Meta-Analysis of Observational Studies. Medicine (Baltimore). 2015;94:e1042.

[11] Wang S, Zhang Y, Mao Z, He X, Zhang Q, Zhang D. A meta-analysis of coffee intake and risk of urolithiasis. Urol Int. 2014;93:220-8.

[12] Liu LH, Kang R, He J, Zhao SK, Li FT, Zhao ZG. Diabetes mellitus and the risk of urolithiasis: a meta-analysis of observational studies. Urolithiasis. 2015;43:293-301.

[13] Ganji-Arjenaki M, Nasri H, Rafieian-Kopaei M. Nephrolithiasis as a common urinary system manifestation of inflammatory bowel diseases; a clinical review and meta-analysis. J Nephropathol. 2017;6:264-9.

[14] Geraghty R, Abdi A, Somani B, Cook P, Roderick P. Does chronic hyperglycaemia increase the risk of kidney stone disease? results from a systematic review and meta-analysis. BMJ Open. 2020;10:e032094.

[15] Thongprayoon C, Cheungpasitporn W, Vijayvargiya P, Anthanont P, Erickson SB. The risk of kidney stones following bariatric surgery: a systematic review and meta-analysis. Ren Fail. 2016;38:424-30.

[16] Upala S, Jaruvongvanich V, Sanguankeo A. Risk of nephrolithiasis, hyperoxaluria, and calcium oxalate supersaturation increased after Roux-en-Y gastric bypass surgery: a systematic review and meta-analysis. Surg Obes Relat Dis. 2016;12:1513-21.

[17] Yu J, Yin B. Postmenopausal hormone and the risk of nephrolithiasis: A meta-analysis. Excli j. 2017;16:986-94.

[18] Gabardo MCL, Wambier LM, Rocha JS, Küchler EC, de Lara RM, Leonardi DP, et al. Association between Pulp Stones and Kidney Stones: A Systematic Review and Meta-analysis. J Endod. 2019;45:1099-105.e2.

[19] Cheungpasitporn W, Rossetti S, Friend K, Erickson SB, Lieske JC. Treatment effect, adherence, and safety of high fluid intake for the prevention of incident and recurrent kidney stones: a systematic review and meta-analysis. J Nephrol. 2016;29:211-9.

[20] Roughley MJ, Belcher J, Mallen CD, Roddy E. Gout and risk of chronic kidney disease and nephrolithiasis: meta-analysis of observational studies. Arthritis Res Ther. 2015;17:90.

[21] Guo ZL, Wang JY, Gong LL, Gan S, Gu CM, Wang SS. Association between cadmium exposure and urolithiasis risk: A systematic review and meta-analysis. Medicine (Baltimore). 2018;97:e9460.

[22] Perletti G, Magri V, Ferraro PM, Montanari E, Trinchieri A. Influence of dietary energy intake on nephrolithiasis - A meta-analysis of observational studies. Arch Ital Urol Androl. 2020;92:30-3.

[23] Rendina D, De Filippo G, D'Elia L, Strazzullo P. Metabolic syndrome and nephrolithiasis: a systematic review and meta-analysis of the scientific evidence. J Nephrol. 2014;27:371-6.

[24] Malihi Z, Wu Z, Stewart AW, Lawes CM, Scragg R. Hypercalcemia, hypercalciuria, and kidney stones in long-term studies of vitamin D supplementation: a systematic review and meta-analysis. Am J Clin Nutr. 2016;104:1039-51.

[25] Kalatharan V, Grewal G, Nash DM, Welk B, Sarma S, Pei Y, et al. Stone Prevalence in Autosomal Dominant Polycystic Kidney Disease: A Systematic Review and Meta-Analysis. Canadian Journal of Kidney Health and Disease. 2020;7.

[26] Lee WS, Kim WS, Lim YH, Hong YC. High Temperatures and Kidney Disease Morbidity: A Systematic Review and Meta-analysis. J Prev Med Public Health. 2019;52:1-13.
